# Chemical Analysis of Nutritional Content of Prickly Pads (*Opuntia ficus indica*) at Varied Ages in an Organic Harvest

**DOI:** 10.3390/ijerph8051287

**Published:** 2011-04-26

**Authors:** Margarita I. Hernández-Urbiola, Esther Pérez-Torrero, Mario E. Rodríguez-García

**Affiliations:** 1 Posgrado en Investigaciones Biomédicas, Universidad Nacional Autónoma de México, Campus Juriquilla, P.O. Box 1–1010, Querétaro, Qro, 76230, México; E-Mail: angel22272@yahoo.com.mx; 2 Departamento de Ciencias de la Salud, Universidad del Valle de México, Campus Querétaro, Querétaro, Qro, 76230, México; 3 División de Investigación y Posgrado, Facultad de Ingeniería, Universidad, Autónoma de Querétaro, Cerro de las Campanas S/N, Querétaro, Qro, 76010, México; 4 Departamento de Nanotecnología, Centro de Física Aplicada y Tecnología Avanzada, Universidad Nacional Autónoma de México, Campus Juriquilla, P.O. Box 1–1010, Querétaro, Qro, 76230, México; E-Mail: marioga@fata.unam.mx

**Keywords:** different maturity stages, powder, nutritional content, *Opuntia*, prickly pads

## Abstract

*Opuntia ficus indica*, also known as prickly pads, are an important part of the human diet and are also used as forage for livestock. This is an interesting vegetable due the environmental conditions in which it grows and its resistance to climatic extremes; however, little is known about its nutritional properties, especially in the later stages of maturity. The objective of this study was to determine the composition of organic prickly pads (*Opuntia ficus indica*) at differing stages of growth maturity. Chemical proximate analysis and mineral constituent analysis at different maturation stages were carried out in this investigation. As a result, older prickly pads were found to be an important source of nutritional components such as calcium.

## 1. Introduction

Organic natural plants are good alternative food sources because they can often contribute to human health and its maintenance. It has been demonstrated in several studies that transgenic and cultivated crops have the potential to directly endanger or damage health because of the pesticide and insecticide treatments used to enhance and protect these crops. Many of these foods are generally associated with health issues or for example, the development of allergies from the toxic effects from fertilizers used to enhance such crops’ nutritional value and also the microbiological safeness of these foods [1].

In Mexico, the cactus *Opuntia ficus indica* has been used since prehispanic times as an important component of the diet and the agricultural economy. They have also been accompanied with other products such as maize, amaranth and agave to name but a few [2]. Prickly pads are grown throughout Mexico and in all North and South American. This cactus grows in many other regions of the World such as Africa, Australia and in the Mediterranean [3]. Cactus pads are commonly called “*nopales*” or “nopalitos” when they are fresh young prickly pads from 3–4 weeks of age. Traditionally “nopales” have been consumed in Mexico and Unites States, using several different preparations or cooking methods. The older stage pads are frequently used as forage, especially when there is shortage of fresh forage due to droughts. This plant is cheap, plentiful and sometimes it has also been used for erosion control [4].

Dietary fiber is composed of several chemical components that are resistant to digestive enzymes such as cellulose, hemicelluloses, pectin, lignin, gums, *etc*. [5]. The fiber content of a food varies according to the species of the plant and its maturity stage. It is noteworthy that plant seeds, berries, fruit skins and the bran layers of cereal grains generally contain large amounts of fiber [6]. The benefits associated with fiber content are well known, especially for the prevention of illnesses such as diabetes, treatment of gastrointestinal disorders, illnesses associated with low dietary fiber intake, reduction of glucose values in the blood, anti-hyperlipidemic and anti-hypercholesterolemic effects [7–10]. Age related differences within the species can be attributed to the type of *Opuntia* and the climatic conditions where the plants grow as a result of factors such as rain precipitation or availability of irrigation.

Previous studies demonstrated that young prickly pads are rich in calcium (Ca) and this increases according to the age of the pads. Most current literature addresses the usefulness of young pads, but does not include data concerning the nutritional and mineral content of prickly pads in their advanced maturity stages [11–13].

*Opuntia* prickly pads are an important source of several nutritional elements like pectin, mucilage and minerals. Currently there is only information related to young stage pads and precious little relating to the nutritional value of older maturity stage pads. The fresh young pads, also known as cladodes, are an excellent source of proteins including essential amino acids, and vitamins. Several studies have reported that high levels of amino acids, especially proline, taurine and serine can also be found in prickly pads [3,13,14]. In contrast, not much information is available about the older prickly pads regarding their amino acid profiles. Also noteworthy is the fact that there is a gap in studies that have already been published regarding.

There is a general consensus among dieticians that diet plays a vital role in the support system of the body. Proper diet provides the strength and nutrition that individuals need to prevent disease, Organic production of food includes cultural, biological, and mechanical practices that foster cycling of resources, promoting ecological balance and conserving biodiversity. Comparative studies of health in populations that habitually consume organically- or conventionally-produced foods clearly indicate the potential health benefits of organic foods. In order to better support the organic food area in the future, more research and of better quality than that which is currently available is necessary [15].

Taking into account the bulk of current findings, the objective of this research was to evaluate the chemical composition of all stages of pads growth in order to evaluate the nutritional properties, and expand the use of prickly pads, taking advantage of production period.

## 2. Materials and Methods

### 2.1. Sample Preparation

The *Opuntia ficus indica* plants or prickly pads were harvested from the field located on a “Los Lores” farm, in Silao, Guanajuato, Mexico. These particular pads were grown without chemical treatment, during the summer (July to August) of 2009. Each sample consisted of 4 kg of organic prickly pads which were collected from several plants in the same sampling areas at different maturity stages. The pads were classified and separated into ten groups according to their age, that being 40, 50, 60, 70, 80, 90, 100, 115, 125, and 135 days, respectively (Table 1). In order to know the age of the pads, the young shoots were marked and followed until 135 age-days. All samples were analyzed in triplicate.

### 2.2. Washed and Dry Vacuum Process

The nopal pads were washed with distilled water and disinfected using commercial 10% sodium hypochlorite solution in order to eliminate microorganisms. The thorns were removed manually and the pads were cut into small slices in order to facilitate the drying process. The prickly pads were then dried using a vacuum system for 12 h at 10^−2^ Torr, and 45 °C, in order to avoid protein and carbohydrate damage. Finally, the prickly pads were pulverized to obtain a powder or flour using a hammer mill (PULVEX 200, Mexico) equipped with a 0.5 mm screen.

### 2.3. Chemical Approximate Analysis

The moisture content of the resulting nopal flour was determined by desiccation at 40 °C for 24 h, according to the 934.01 method as described in the Association of Official Analytical Chemists (AOAC) techniques [16]. The chemical analyses of the prickly pads were carried for ten groups at different maturity stages. This included protein, carbohydrates, fat, non organic components (ash) and humidity. Analyses were done in triplicate according to the AOAC techniques [16]. The evaluation of the flours was performed using specific methods for different components.

Mineral ash content was evaluated with the 942.05 method [16], using 2 g samples, determined at 550 °C for 24 h in order to remove organic material. The samples were placed in shallow, relatively broad ashing dishes that had been ignited. The samples were then cooled in a desiccator, and weighed once they reached room temperature.

Nitrogen (N) concentration was ascertained by applying the Kjeldahl method 2001.11 of AOAC [16], using a 0.5 g sample. The carbohydrate free nitrogen extract, was determined by calculating the differences in 100 g of all the components using AOAC offcial method 986.25 1986 [16]. Crude fiber was determined according to the 991.42 and 993.19 AOAC methods. Fat was analyzed by petroleum ether extraction using a Soxhlet apparatus according to the 920.39 AOAC methods [16].

### 2.4. Atomic Absorption Spectroscopy (AAS)

The Ca, Mg, K, and Na contents were determined using the dry-ashing procedure 968.08 [16]. The Ca, Mg, K, Na ions concentrations were measured with a double beam atomic absorption spectrometer, (Analyst 300, Perkin Elmer, USA). The organic components were previously eliminated at 550 °C for 24 h.

### 2.5. Mass Spectrometry ICP-MS

The mineral elements lithium (Li), vanadium (V), phosphorus (P), manganese (Mn) iron (Fe), cobalt (Co), arsenic (As), zinc (Zn), selenium (Se), cadmium (Cd), and thallium (Tl) of prickly pads powder were quantified by means of ICP-MS mass spectrometry following the U.S. Environmental Protection Agency Guidelines [17]. The tests were carried out following the AOAC method (984.27) [16] using a Thermo Jarrel Ash, Model IRIS/ICP ICP Spectrophotometer. The IRIS Optical Emission Spectrometer is inductively coupled argon plasma, optical emission spectrometer which uses Echelle optics and a unique charge injection device, solid state detector in order to provide complete and continuous wavelength coverage over the typical analytical wavelength range. The IRIS is equipped with Radial Plasma for the widest range of applications. The unit includes ICP, chiller, power supply, and autosampler, computer with software loaded with no software media, service and operation manual.

### 2.6. Statistical Analysis

Data, based on three replicates were subjected to analysis of variance. Standard deviation of each individual nutrient regarding maturity stage mean was computed and variations between maturation stages were evaluated by using Tukey test at a 5% level of probability (P = 0.05). All statistical data was calculated using stat graphics.

## 3. Results and Discussion

Prickly pads powders present a large decrease in humidity in relation to the fresh prickly pads which is inherent to the vacuum drying process (Table 1); this procedure prevents the proliferation of microorganisms to render the pads edible [18].

The fat content of the powders decreased as a function of age and no direct relationship with age-days was observed [R^2^ = 0.3319 (Table 1)]. The decreases noticed were perhaps due to physiological changes or differences in climatic condition such as precipitation or irrigation where the plants were grown [6]. For protein, which was low for all studied ages and similar to those of other vegetables [19], no related changes were observed (Table 1). Findings suggest that physical conditions such as water availability, temperature, and light/dark periods are primarily implicated in protein synthesis. Several studies have demonstrated that protein synthesis increases as a cellular protection when the soil is too acid or saline [20–22].

Carbohydrate content of old prickly pads showed significant increases from 42.94 mg/g at day 40 to 60.77 mg/g at day 135. Furthermore, they also showed a direct relationship (R^2^ = 0.5446) pertaining to age. As with all vegetables, carbohydrates are the main component in prickly pads [13].

The ash content of minerals Ca and Fe (Table 2) increased in 40 to 135 days old samples, whereas P, Mn and Zn did not show any age related changes. The linear regression analysis showed a positive relationship (R^2^ = 0.7158) in regard to prickly pads age. The vanadium, cobalt and selenium levels showed minimal changes in relation to age, with maximal content at 80, 90, and 100 age-days. For the lithium, arsenic, cadmium and thallium content, the data revealed minimal content, without important changes associated with ages. These findings suggest that the prickly pads powders might be a complement to the daily diet due to their essential microminerals content (Tables 2 and Table 3). It is suggested that some elements of ash chemical composition depend on different factors, such as pH, water availability, soil texture and composition where the nopal grow. These results are in line with previous studies which reported that the main minerals contained in prickly pads are in the form of carbonates, chlorides, sulfates and phosphates [23,24].

The data collected herein suggests that the best age to harvest and consume pads is at 135 days because of the elevated calcium content, which has applications in the prevention and treatment of diseases such as osteoporosis. The results also indicate that nopal also has potential for the treatment of diabetes due to its fiber content.

Therefore, the consumption of prickly pads in advanced maturity stages as a powder should be promoted, which may encourage increased use and marketing of this plant at its advanced stages of maturity. It can also be used in the production of cosmetics due to its consistency, which is based on the fiber composition [19,25]. Several researchers have observed decreases in the LDL-cholesterol and triglycerides when individual dietary intakes were supplemented with prickly pads at 40–50 age-days [6,7]. The crude fiber showed a positive relationship related to the age, however, the soluble dietary fiber tended to have negative relationship, suggesting than older nopal is better source of insoluble fiber [8,9,19], so prickly pads can be a rich source of soluble fiber during younger ages and show increased insoluble fiber content at older ages.

Because nutritional deficiencies are currently widespread in many poor areas of the World, special attention should be focused on inexpensive solutions, such as nopal powders, in order to take advantage of all of its production periods. Nopal powders can be an economic alternative when used as a dietary supplement in all seasons, without the need for fresh nopal. The dried products represent certain advantages for transport and preservation for prolonged periods in optimal conditions to ensure maximum nutritional quality and availability. A noteworthy observation made from this study is the recommended proposal of increasing daily intake of nopal flour produced from older pad as it is related to increased Ca content found in the advanced maturity stages. In general, it was concluded that the nutrimental value are associated with the time of harvest and therefore the maturation stages of the prickly pads [19]. The results herein indicate that powder from prickly pads at more mature stages can be a natural source of calcium and should be included in human diets. In addition, others authors have reported the phenols and flavonoids composition in *Opuntia spp*. in order to understand the antioxidant properties, which complements the information concerning nutritional and nutraceutical values of prickly pads [26–28].

## 4. Conclusions

The chemical and mineral compositions of several maturity stages in the prickly pads (*Opuntia ficus indica*) samples were different according to age-days. The best stage of prickly pads for calcium content was found to be 135 days-age. The results indicate that older prickly pads powder can be a good source of calcium for populations where the availability of dairy products is complicated, and also in people with difficulties in digesting dairy products. Current findings could promote the consumption of prickly pads according to the health benefit associated to the composition of young and older prickly pads.

## Figures and Tables

**Table 1.** Chemical composition of dehydrated prickly pads (*Opuntia ficus indica*, 100 g of sample).

**Prickly pads age (days)**	**Prickly pads weight (g)**	**Moisture (g)**	**Ash (g)**	**Fat (g)**	**Crude Fiber (g)**	**Protein (g)**	**Carbohydrates(g)**
40	100	5.03 cd	17.65 a	2.16 e	11.00 a	7.07 b	42.94 a
50	150	8.81 e	19.59 b	2.37 f	13.26 b	8.99 e	53.04 b
60	200	5.43 d	20.64 c	2.38 f	16.14 c	8.39 d	53.01 b
70	250	4.85 bcd	21.09 d	1.62 bc	19.03 d	8.92 e	55.53 d
80	300	4.36 ab	21.64 e	1.53 ab	18.73 d	7.25 b	53.53 bc
90	350	4.81 bc	21.92 f	1.50 ab	19.12 d	7.78 c	55.15 cd
100	400	4.08 a	22.80 g	1.42 a	20.11 de	8.29 d	56.73 d
115	450	4.58 abc	22.91 h	1.72 c	21.48 e	8.48 d	59.20 e
125	500	4.35 ab	20.91 i	1.70 c	19.85 d	5.85 a	52.67 b
135	550	4.18 a	24.30 j	1.87 d	23.33 f	7.07 b	60.77 e

Results for each component *vs*. age followed with the same letter in the column, were not significantly (P < 0.05) different.

**Table 2.** Mineral composition of prickly pads powder at different maturational stage age-days.

**Maturity stage (age-days) (mg/g)**										

**Mineral**	**40**	**50**	**60**	**70**	**80**	**90**	**100**	**115**	**125**	**135**
Phosphorus	2.59 a	4.26 b	4.48 b	4.39 b	4.06 b	4.60 b	3.77 abc	5.00 c	3.15 a	3.94 c
Manganese	0.29	0.06 a	0.07 a	0.05 a	0.08 a	0.05 a	0.05 a	0.05 a	0.03 a	0.08 a
Iron	0.09 a	0.09 a	0.10 a	0.12 b	0.12 b	0.134 b	0.132 bc	0.14 c	0.16	0.22
Zinc	0.08	0.06	0.04	0.08	0.03	0.04	0.06	0.05	0.04	0.06
Magnesium	8.80 a	10.60 e	11.20 f	11.5 g	10.2 d	12.00 h	11.00 f	11.95 h	8.95 b	9.55 c
Calcium	17.95	22.10 a	24.00 a	27.00 b	28.35 bc	28.65 bc	29.20 bc	29.15 c	30.70 c	34.40
Potassium	55.20 a	64.75 bc	70.90 d	68.50 bcd	72.20 d	69.70 cd	69.95 d	71.45 d	51.80 a	63.35 b
Sodium	0.30	0.40	0.30	0.35	0.55	0.35	0.20	0.50	0.20	0.30

Results for each mineral *vs*. age followed with the same letter in the line, were not significantly (P < 0.05) different.

**Table 3.** Minor mineral composition, of prickly pads powder at different maturational stage, age-days.

**Maturity stage (age-days) (mg/100g)**										
**Mineral**	**40**	**50**	**60**	**70**	**80**	**90**	**100**	**115**	**125**	**135**
Lithium	0.80	0.23	1.0	1.4	0.99	0.07	0.55	0.18	0.19	0.29
Vanadium	0.33 a	1.43 b	1.61 b	1.66 b	2.27 b	2.19 b	2.07 ab	0.68 a	0.94	0.79 a
Cobalt	0.16 a	0.13 ab	0.15 abc	0.14 bde	0.18 c	0.12 d	0.10 de	0.15 de	0.11 e	0.21
Arsenic	0.14	0.05	0.04	0.00	0.007	0.00	0.04	0.002	0.00	0.00
Selenium	0.38	0.14	0.13	0.001	0.05	0.13	0.09	0.005	0.00	0.01
Cadmium	0.05	0.00	0.03	0.08	0.00	0.01	0.00	0.00	0.00	0.06
Thallium	0.07	0.06 a	0.06 a	0.06 a	0.06 a	0.06 a	0.06 a	0.07 a	0.82	0.06

Results for each mineral *vs*. age followed with the same letter in the line, were not significantly (P < 0.05) different.
